# Activation of immune receptor Rx1 triggers distinct immune responses culminating in cell death after 4 hours

**DOI:** 10.1111/mpp.12776

**Published:** 2019-01-30

**Authors:** Marijn Knip, Manon M.S. Richard, Lisa Oskam, Hylco T.D. van Engelen, Thomas Aalders, Frank L.W. Takken

**Affiliations:** ^1^ Molecular Plant Pathology University of Amsterdam, SILS Sciencepark 904 Amsterdam 1098SM the Netherlands

**Keywords:** DNA damage, ETI, immune receptor, NLR, plant immunity, ROS, Rx1

## Abstract

Intracellular nucleotide‐binding leucine‐rich repeat (NLR)‐type immune receptors are a fundamental part of plant immune systems. As infection occurs at foci, activation of immune responses is typically non‐uniform and non‐synchronized, hampering the systematic dissection of their cellular effects and determining their phasing. We investigated the potato NLR Rx1 using the CESSNA (Controlled Expression of effectors for Synchronized and Systemic NLR Activation) platform. CESSNA‐mediated *Potato virus X* coat protein (CP) expression allowed the monitoring of Rx1‐mediated immune responses in a quantitative and reproducible manner. Rx1 was found to trigger a reactive oxygen species (ROS) burst and ion leakage within 1 h and a change in autofluorescence within 2 h after the induction of CP production. After 2 h, *HIN1* expression was increased and single‐stranded DNA (ssDNA) damage and loss of cellular integrity became apparent, followed by double‐stranded DNA (dsDNA) damage after 3 h and increased *PR‐1a*, *LOX*, *ERF1* and *AOX1B* expression and cell death at 4 h. Nuclear exclusion of Rx1 resulted in increased basal levels of ROS and permitted Rx1 activation by an Rx1‐breaking CP variant. In contrast, nuclear‐targeted Rx1 showed diminished basal ROS levels, and only avirulent CP could trigger a compromised ROS production. Both nuclear‐excluded and nuclear‐targeted Rx1 triggered a delayed ion leakage compared with non‐modified Rx1, suggesting that ion leakage and ROS production originate from distinct signalling pathways. This work offers novel insights into the influence of Rx1 localization on its activity, and the interplay between Rx1‐triggered processes.

## Introduction

The specificity of plant immune systems to perceive pathogen attack is conferred by a combination of cell surface and intracellular receptors. Immune receptors located in the plasma membrane recognize extracellular pathogen‐derived triggers to activate a first line of defence. The downstream immune outputs of some membrane‐localized immune receptors have been well described, such as the recognition of bacterial flagellin or the derived flg22 peptide by FLS2 (Boller and Felix, 2009; Gomez‐Gomez and Boller, 2000). Within minutes after receptor activation, FLS2 triggers various cellular responses, including a rapid burst of calcium influx and the production of reactive oxygen species (ROS). In the hours after FLS2 activation, antimicrobial compounds are produced, cell walls are reinforced by callose deposition (apparent after 6–8 h) and programmed cell death sometimes occurs (typically days after activation) (Luna *et al*., 2011; Nguyen *et al*., 2010; Segonzac *et al*., 2011). The dissection of outputs triggered by plasma membrane‐localized receptors has been facilitated by the fact that they can be uniformly activated by exogenously applied elicitors and availability of widely adapted tools (Chakravarthy *et al*., 2009; Lloyd *et al*., 2014; Nguyen *et al*., 2010; Segonzac *et al*., 2011).

Intracellular pathogen recognition—typically by the perception of microbial effector proteins—is mediated by cytosolic immune receptors. The vast majority of these receptors belong to the nucleotide‐binding leucine‐rich repeat (NLR) family. Their activation cannot be triggered by externally applied molecules, as the perception of effectors relies on pathogen‐mediated delivery and/or host cell uptake (Di *et al*., 2016; Dodds and Rathjen, 2010). Therefore, the study of the precise sequence of cellular events following intracellular immune receptor activation is challenging. As a result of pathogen infection strategies, the responses triggered by these immune receptors are typically not synchronous across infected tissues, resulting in a heterogeneous readout. Furthermore, following pathogen invasion, both cell surface and intracellular immune receptors are activated, complicating the exclusive monitoring of readouts from the latter. These aspects have hindered the systematic dissection of immune outputs mediated by intracellular receptors. Yet, immune receptors and their respective triggers have been identified in a large variety of plant–pathogen systems, signifying the importance of a system to study and compare their responses. This article describes the detailed and chronological study of immune responses induced by the Rx1 immune receptor using an experimental system called CESSNA (Controlled Expression of effectors for Synchronized and Systemic NLR activation), which enables the study of immune responses induced by immune receptors at a high resolution.

Using *Nicotiana benthamiana* as a heterologous host, potato Rx1 has been the focus of many NLR studies, expanding our molecular understanding of NLR activation, dynamics and structure (Fenyk *et al*., 2015; Rairdan and Moffett, 2006; Ritter *et al*., 1991; Slootweg *et al*., 2010; Tameling *et al*., 2010). On effector recognition, NLRs typically initiate a multitude of defence responses, which often culminate in the death of infected cells, called the hypersensitive response (HR). Rx1 recognizes the coat protein (CP) of *Potato virus X* (PVX) and confers ‘extreme resistance’ to this virus, which is defined as immunity without triggering cell death (Kohm *et al*., 1993). However, when the *CP* gene is overexpressed, Rx1 triggers HR (Bendahmane *et al*., 1999). An extensive set of Rx1 mutants is available, and versions of Rx1 with distinct subcellular localizations have been created to gain insights into Rx1 function (Bendahmane *et al*., 2002; Fenyk *et al*., 2015, 2016; Moffett *et al*., 2002; Rairdan and Moffett, 2006; Slootweg *et al*., 2010; Takken and Goverse, 2012; Townsend *et al*., 2018). Fusion of a nuclear export signal (NES) or a nuclear localization signal (NLS) to Rx1 revealed that CP recognition occurs exclusively in the cytosol (Slootweg *et al*., 2010). Moreover, to trigger full resistance, Rx1 needs to be able to dynamically distribute between the cytosol and the nucleus (Slootweg *et al*., 2010). Both NES and NLS Rx1 fusions can trigger cell death when exposed to the CP, but can no longer contain viral spread (Slootweg *et al*., 2010). That Rx1 can bind DNA *in vitro*, and needs both a nuclear and cytosolic localization, is interesting in the light of the occurrence of DNA damage during the onset of immunity (Fenyk *et al*., 2015; Song and Bent, 2014; Song *et al*., 2011; Yan *et al*., 2013). Immune signalling itself can induce DNA damage via an unknown mechanism (Rodriguez *et al*., 2018; Yan *et al*., 2013).

Rx1 activation was synchronized using a dexamethasone (DEX)‐inducible CP construct in *N. benthamiana* expressing *Rx1* constructs. The onset, amplitude and duration of distinct immune readouts induced by controlled Rx1 activation were monitored. Where applicable, assays were adjusted for use with a plate reader to allow the generation of quantitative data on Rx1‐triggered responses with a high spatial resolution.

## Results

### CESSNA: a system to study synchronized Rx1 activation

**Figure 1:**
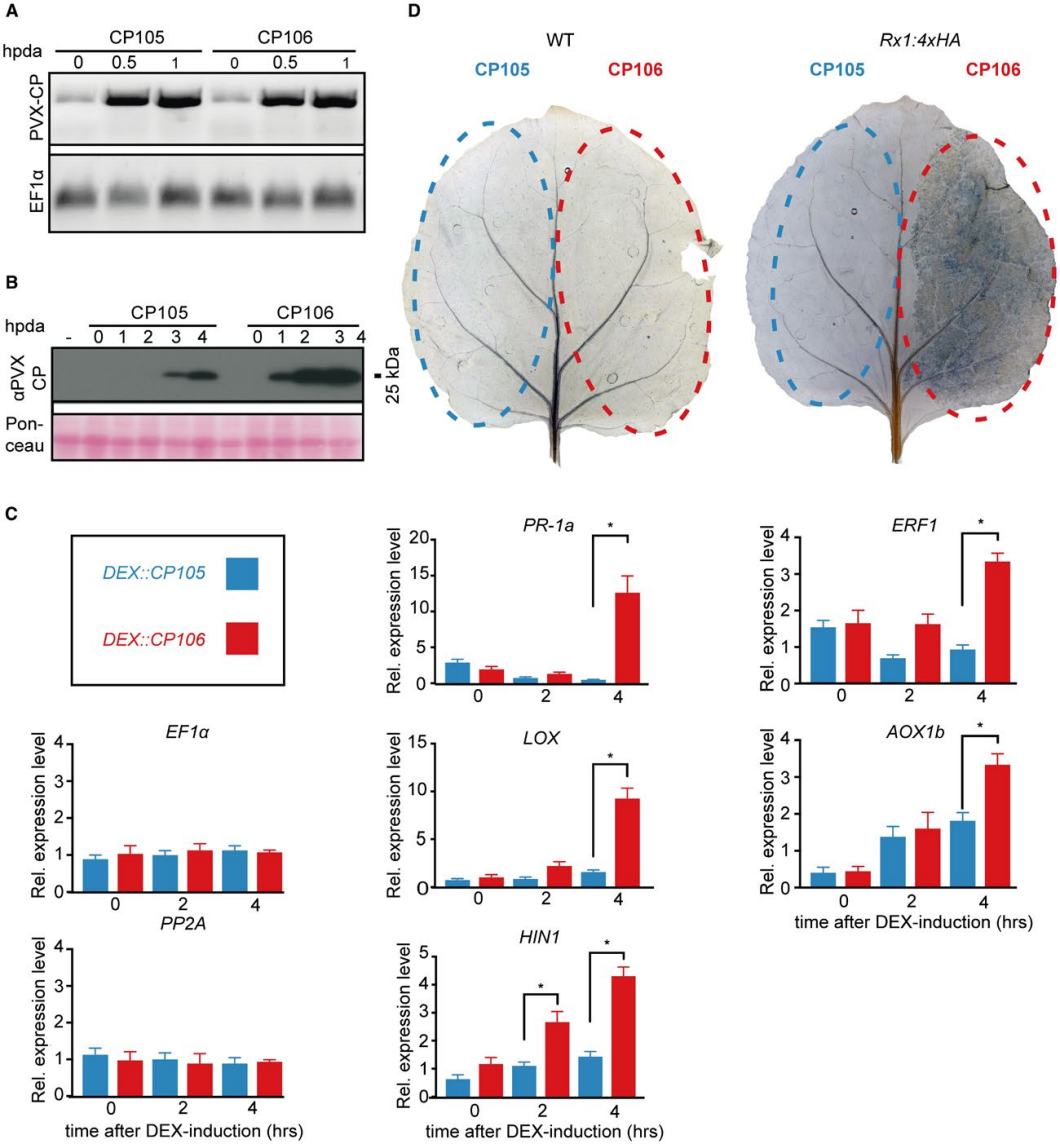
Verification of CESSNA [Controlled Expression of effectors for Synchronized and Systemic NLR (nucleotide‐binding leucine‐rich repeat) Activation]: defence gene expression, cell death and coat protein (CP) accumulation. (A) *Potato virus X* (PVX) CP accumulation in wild‐type (WT) plants expressing *DEX::CP105* or *DEX::CP106. *Semi‐quantitative reverse transcription‐polymerase chain reaction (RT‐PCR) at 0, 0.5 and 1 h after dexamethasone (DEX) application (hpda); expression of *EF1α* serves as an internal control (bottom panel). (B) Western blot showing PVX CP accumulation in WT plants transiently expressing *DEX::CP105* or *DEX::CP106 *at 0, 1, 2, 3 and 4 hpda. Blots were probed with horseradish peroxidase (HRP)‐conjugated goat anti‐rabbit immunoglobulin G (IgG) secondary antibody. (C) Quantitative RT‐PCR analysis of defence response and hypersensitive response marker genes. Expression pattern of PR‐1a, LOX, HIN1 ERF1 and AOX1b after DEX induction of *CP105* and *CP106* in *Rx1:4xHA* *N. benthamiana*. Data are means ± standard error (SE), normalized by *EF1α* and *PP2A* expression. Asterisks indicate significant differences by one‐way analysis of variance (ANOVA) (*P* < 0.0001). (D) Trypan blue visualizes cell death. WT and *Rx1:4xHA* leaves infiltrated with *Agrobacterium tumefaciens *carrying *DEX::CP105* and *DEX::CP106* constructs. Two days after infiltration, leaves were brushed with 20 µm DEX and stained the following day.

The CESSNA system was developed to synchronize CP‐triggered immune activation of Rx1 to study and quantify its distinct immune readouts. Transgenic *N. benthamiana* plants, stably expressing *Rx1:4xHA*, were used for the transient expression of inducible constructs that allow controlled CP production (Fig. 1) (Lu *et al*., 2003).

CP106, a CP derived from an avirulent PVX strain recognized by Rx1, was used to trigger Rx1‐mediated immune activation. CP105, a non‐Rx1‐recognized CP from a virulent virus, was used as a negative control (Goulden and Baulcombe, 1993; Jones *et al*., 1999). The CP coding sequences were cloned in a DEX‐inducible expression vector. After brushing DEX onto agroinfiltrated *Rx1:4xHA N. benthamiana*, the *CP106*‐expressing leaf sectors showed visual tissue collapse at approximately 4 h after DEX application (hpda), whereas no collapse was observed for *CP105*‐expressing sectors (Video S1, see Supporting Information), designating the time frame of obtaining relevant immune outputs to 4 hpda.

The kinetics of CP transcript and protein accumulation after DEX induction were studied by quantitative reverse transcription‐polymerase chain reaction (RT‐PCR) and western blotting. *CP* expression analysis revealed small amounts of *CP* transcript before DEX induction, and a strong accumulation at 0.5 hpda (Fig. 1A). After DEX application, CP protein was detected at 2 hpda for CP106 and at 3 hpda for CP105 (Fig. 1B). No CP was detected before DEX application (Fig. 1B).

To monitor the expression of defence‐related marker genes in this time frame, quantitative RT‐PCR analysis was performed. On *Agrobacterium* infiltration and brushing of DEX onto the transformed leaves of *Rx1:4xHA* plants, CP106 production triggered *PR‐1a*, *LOX*, *HIN1*, *ERF1 *and *AOX1b *defence gene expression and cell death, whereas CP105 did not (Fig. 1C,D). *PR‐1a*, *LOX *and *HIN1 *are marker genes for salicylic acid (SA) defence activation, jasmonic acid biosynthesis and cell death, respectively (Garcia‐Marcos *et al*., 2013; van Loon *et al*., 2006; Pontier *et al*., 1999). *ERF1* is an ethylene‐responsive transcription factor and *AOX1b* is an oxidase shown to be involved in antiviral defence (Huang *et al*., 2016; Lee *et al*., 2011). For *HIN1*, a significant change in gene expression was observed already at 2 hpda, whereas the other genes showed a significant difference only at 4 hpda. The transcriptional responses are not directly linked to the death of cells, as the expression of the marker genes *EF1α* and *PP2A* remained constant over the 4‐h time frame (Fig. 1C). Furthermore, the expression of *PR1b* and *PR2b* (both pathogenicity‐related genes) and of mitogen‐activated kinase *MAP3Ka* was not significantly different in CP106‐ relative to CP105‐expressing leaves (Fig. S1A, see Supporting Information). These data indicate that a subset of the major defence signalling pathways is activated following Rx1 action. Taken together, these results reveal that CESSNA is a suitable system to investigate the onset, amplitude and duration of events induced by activated Rx1.

### Rx1 activation triggers a ROS burst

**Figure 2:**
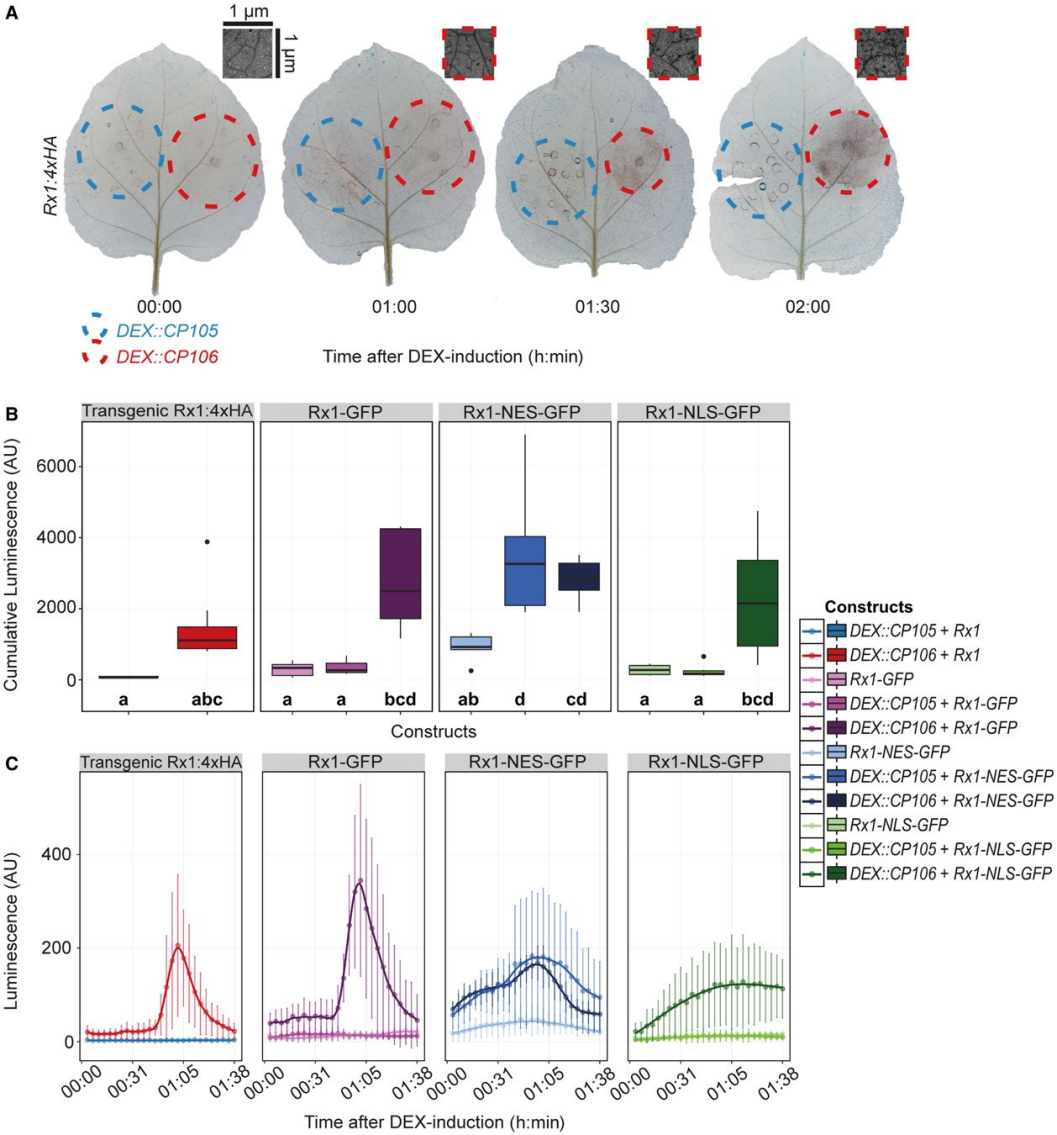
Activation of Rx1 triggers a rapid reactive oxygen species (ROS) burst. (A) 3,3′‐Diaminobenzidine (DAB) staining of *DEX::CP105* and *DEX::CP106 *agroinfiltrated *Rx1:4xHA* leaves at three time points after dexamethasone (DEX) induction. Inset shows a close‐up of a representative *CP106/Rx1* sector. (B) Box‐and‐whisker plots showing the cumulative production of ROS, measured using a plate reader‐based assay in arbitrary units (AU). Outliers are indicated by individual dots. Left panel shows *Rx1:4xHA* plants in which *DEX::CP105* or *DEX::CP106 *was expressed. The other three panels show the co‐expression of the coat protein (*CP*) constructs with green fluorescent protein (GFP)‐tagged Rx1 wild‐type or a nuclear‐localized (Rx1‐NLS) or nuclear‐excluded (Rx1‐NES) version. Letters depict significantly different (*P* < 0.05) categories, as determined by Tukey’s honestly significant difference (HSD) test. Each column consists of cumulative measurements of 9–15 discs. (C) Kinetics of ROS production over time following DEX treatment of the samples depicted in (B).

Little is known about the dynamics of ROS production after NLR activation. In this context, ROS production following Rx1 activation was investigated. To study ROS production, 3,3′‐diaminobenzidine (DAB) staining and luminol‐based plate reader experiments were performed. DAB staining was performed by infiltrating DAB staining solution into sectors of the leaf that had been agroinfiltrated previously to carry DEX‐controlled *CP* constructs (Fig. 2A). Subsequently, DEX was brushed onto the leaf surface and the leaves were collected and de‐stained at 0, 1, 1.5 and 2 hpda (Fig. 2A). DAB staining only became visible in CP106 sectors at 1.5 and 2 hpda (Fig. 2A).

To quantitatively analyse ROS production, leaf discs were collected 1 day after agroinfiltration and placed in water overnight. On the following day, individual leaf discs were placed in a white 96‐well plate. Subsequently, DEX was added to induce *CP* expression, and peroxidase and luminol were supplemented to monitor ROS generation. ROS production was quantified in a BioTek Synergy plate reader (Winoosk, Vermont, USA) by recording the luminescence over time (Fig. 2B,C). The experimental set‐up and the ability of the leaf discs to mount a ROS burst were validated by flg22 treatment, which resulted in a typical flg22‐induced burst peaking at 25 min (Fig. S2, see Supporting Information) (Chinchilla *et al*., 2007). To monitor Rx1 responses, *Rx1:4HA* plants in combination with DEX‐inducible CP constructs and wild‐type (WT) plants agroinfiltrated with both *Rx1* and DEX‐inducible *CP* constructs were used (Fig. 2B,C). As a negative control, CP105 was used in combination with different *Rx1* constructs (Fig. 2B,C). Recordings were limited to 2 hpda, as leaf disc homeostasis deteriorated after this time point, leading to non‐immune‐related ROS production (Smith and Heese, 2014). Rx1 activation triggered a ROS burst with a peak at approximately 1 hpda in *Rx1:4xHA* plants expressing agroinfiltrated *DEX::CP106*, and in WT plants co‐expressing agroinfiltrated *Rx1:GFP* and *DEX::CP106* (Fig. 2B, left panel). The ROS burst triggered by Rx1 is different from the flg22‐induced burst, as flg22 triggers a sharp burst at around 30 min after treatment, whereas Rx1 induction produces a wider ROS peak with a lower amplitude showing a maximum around 1 hpda (Figs 2C and S2).

Rx1 requires both a nuclear and cytosolic localization to be fully functional (Slootweg *et al*., 2010). To investigate the consequences of Rx1 localization on the dynamics of ROS production, we fused Rx1 to either an NES (Rx1‐NES) or an NLS (Rx1‐NLS). We found that both Rx1‐NES and Rx1‐NLS versions were able to induce ROS production (Figs 2B,C and S3, see Supporting Information). However, ROS curves triggered by Rx1‐NES, and especially Rx1‐NLS, did not result in distinct peaks, as were found for Rx1:4xHA and Rx1‐GFP. This difference is not caused by differences in Rx1 protein accumulation, as both the NLS and NES fusions accumulated to similar levels to the Rx1‐GFP fusion (Fig. S3). In the presence of CP106, Rx1‐NLS induced a slow and moderate production of ROS without a clear peak (Fig. 2B,C). Rx1‐NES, however, triggered ROS accumulation in the presence of either CP105 or CP106 (Fig. 2B,C). In fact, *Rx1‐NES* expression alone resulted in slightly elevated ROS levels. Therefore, although CP105 and CP106 were both able to trigger Rx1‐NES‐mediated ROS production, the peak in ROS production was lower and basal levels were elevated. We found that Rx1 cellular localization affects the dynamics of ROS production after immune activation, and that the presence of Rx1 in both the cytoplasm and nucleus is required to produce a defined ROS burst.

### DNA damage occurs after activation of Rx1

The onset of plant immunity is associated with increased levels of DNA damage (Rodriguez *et al*., 2018; Yan *et al*., 2013). We therefore applied CESSNA to study DNA damage following Rx1 activation. Two methods were employed to monitor DNA damage over time. First, the terminal deoxynucleotidyl transferase dUTP nick‐end labelling (TUNEL) assay was adapted to quantitatively assess DNA damage in a 96‐well plate reader set‐up. The TUNEL assay labels single‐stranded (ss) and double‐stranded (ds) breaks by the incorporation of a fluorescein‐labelled nucleotide at DNA termini (Gavrieli *et al*., 1992). Second, a single‐nucleus gel electrophoresis (comet assay) method was applied (Menke *et al*., 2001), which enabled the differentiation between ssDNA and dsDNA breaks. These experiments were performed in *Rx1::4xHA* plants agroinfiltrated with either *DEX::CP105 *or *DEX::CP106, *and DEX induction was performed by brushing DEX on infiltrated leaves.

**Figure 3:**
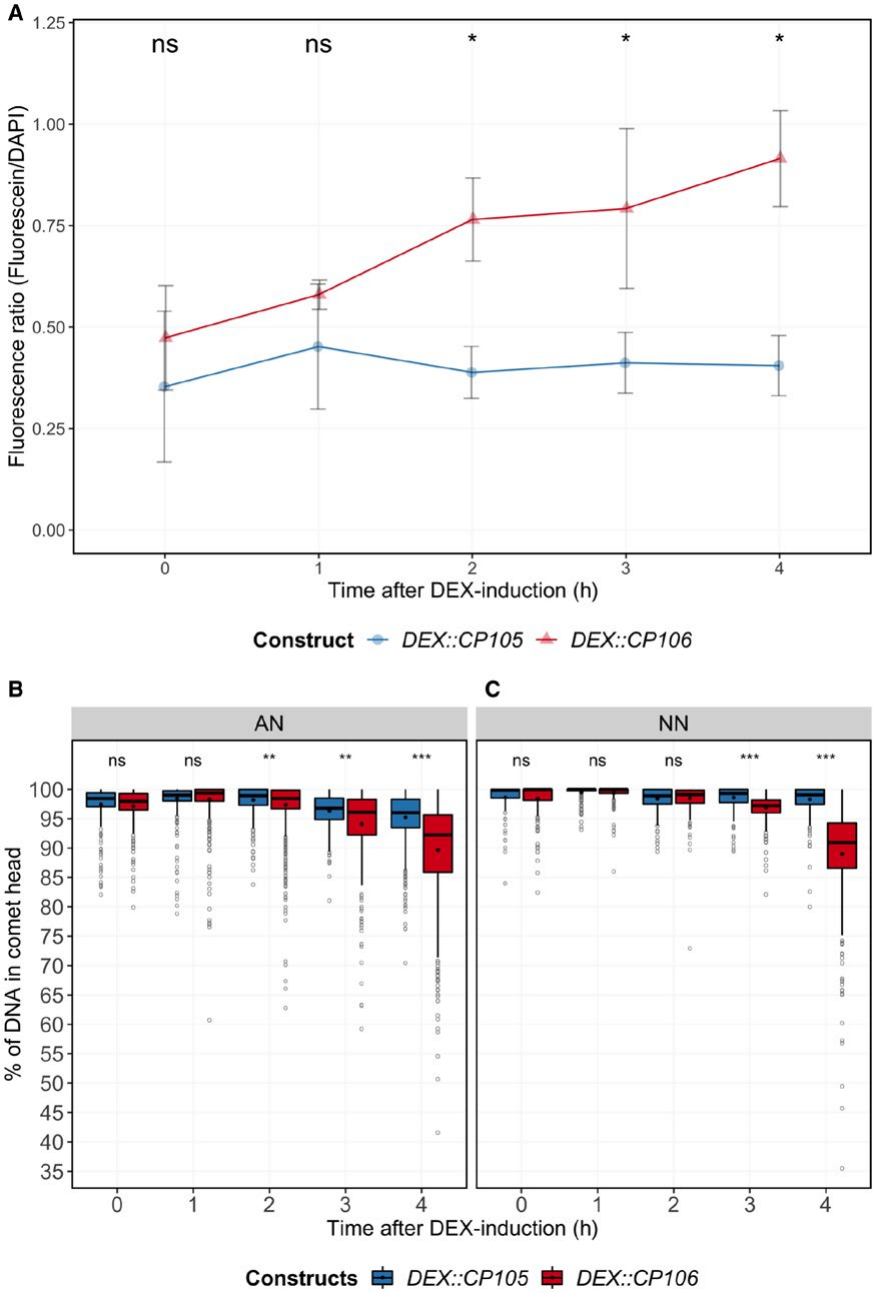
Rx1 activation triggers single‐stranded DNA (ssDNA) and double‐stranded DNA (dsDNA) breaks. (A) Terminal deoxynucleotidyl transferase dUTP nick‐end labelling (TUNEL) assay revealing DNA damage in *DEX::CP105* and *DEX::CP106 *agroinfiltrated *Rx1:4xHA* leaves following dexamethasone (DEX) application. Fluorescein‐labelled UTP is incorporated at sites of DNA damage. To allow sample comparison, the fluorescence of fluorescein (proxy for DNA damage) was divided by that of 4′,6‐diamidino‐2‐phenylindole (DAPI)‐stained DNA (proxy for DNA amount). Each value consists of three to six samples and each sample was obtained from separate plants. Statistical analysis using Wilcoxon non‐parametric test: ns, no significant difference; *P < 0.05. Comet assay showing DNA damage in plant nuclei with induced *DEX::CP105* and *DEX::CP106* constructs in a 4‐h time course, using (B) high‐alkaline (AN) buffer showing ssDNA and dsDNA breaks, and (C) using high‐salt (NN) buffer revealing only dsDNA breaks. Nuclei (comets) were counted and visualized using a box‐and‐whisker plot. The number of nuclei per sample can be found in Table S1. Statistical analysis using Wilcoxon non‐parametric test: ns, no significant difference; **P < 0.001; ***P < 0.0001.

For the TUNEL assay, nuclei were extracted from leaf material at four different time points after DEX administration: 0, 1, 2, 3 and 4 hpda. Nuclear DNA was stained with 4′,6‐diamidino‐2‐phenylindole (DAPI) to quantify the total amount of DNA in the samples. DNA damage was visualized by monitoring the amount of incorporated fluorescein‐labelled nucleotides using the TUNEL procedure. Nuclear suspensions of the various samples that were both stained and labelled were transferred to 96‐well plates and measured in a plate reader. The resulting fluorescein/DAPI ratios were calculated and used as a proxy for the relative amount of DNA damage (Fig. 3A). The ratio increased from 0.473 to 0.915 in the presence of CP106, indicating DNA damage, whereas CP105 induction did not result in elevated levels (0.353–0.405) (Fig. 3A). Values were significantly different between CP105 and CP106 samples at 2, 3 and 4 hpda (Wilcoxon signed rank test, *P* < 0.05).

Comet assays use the mobility of DNA as a representation of the extent of ss or ds breaks at the single‐nucleus level. Nuclear extractions were performed at 0, 1, 2, 3 and 4 hpda (Table S1, see Supporting Information). Nuclei were embedded in low‐melting‐point agarose and subjected to treatment with either high‐salt (NN) or high‐pH (AN) buffer prior to electrophoresis (Menke *et al*., 2001). The NN buffer does not denature dsDNA and therefore only allows the migration of fragments originating from ds breaks. AN buffer denatures dsDNA and facilitates the migration of fragments resulting from both ss and ds breaks. As a negative control, DEX‐inducible *GFP* was included to assess the impact of the experimental set‐up on DNA integrity (Fig. S4, see Supporting Information). At 2 hpda (AN buffer) and 3 hpda (NN buffer), DNA fragmentation was significantly higher (Wilcoxon signed rank test, *P* < 0.01) in CP106 relative to CP105 samples (Fig. 3B,C). In addition, the amount of DNA damage continued to increase over time in CP106 material, whereas it remained constant in CP105 samples (Fig. 3B,C) and in the green fluorescent protein (GFP) control (Fig. S4). In conclusion, Rx1‐induced DNA damage commences at approximately 2 hpda and increases at later time points. The data suggest that ss breaks precede ds breaks on Rx1 activation.

### Cell membrane integrity decreases after Rx1 activation

**Figure 4:**
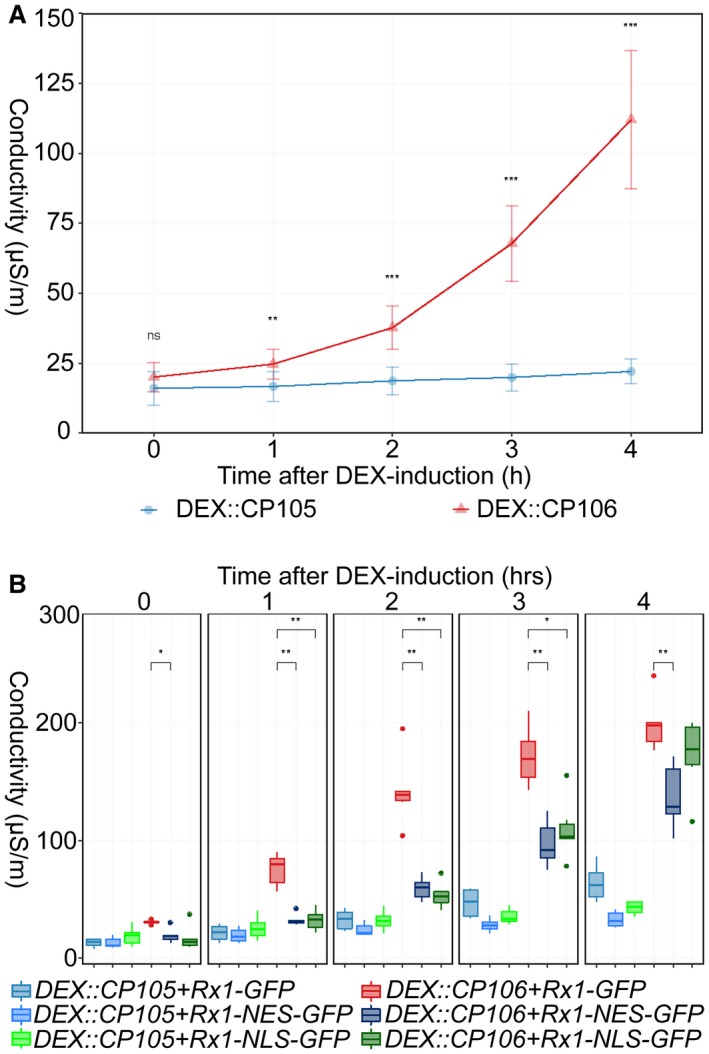
Rx1 activation induces ion leakage. (A) Ion leakage in *Rx1::4HA *leaf discs after dexamethasone (DEX) induction of *DEX::CP105* and *DEX::CP106 *constructs. Values represent measurements from nine leaf discs from the same leaf. This is a representative experiment that was repeated more than three times with similar results. Statistical analysis using Wilcoxon non‐parametric test: ns, no significant difference; **P < 0.01; ***P < 0.001. (B) Rx1‐, Rx1‐NLS‐ and Rx1‐NES‐triggered ion leakage after DEX induction of *CP105* and *CP106*. Discs were obtained from wild‐type plants that had been agroinfiltrated to produce green fluorescent protein (GFP)‐tagged Rx1 variants with both coat protein (CP) constructs. The five panels contain hourly measurements from one time point each, represented in a box plot. Each value represents measurements from six leaf discs from the same leaf. This is a representative experiment that was repeated more than three times with similar results. Statistical analysis using Wilcoxon non‐parametric test: ns, no significant difference; *P < 0.05; **P < 0.01.

Electrolyte leakage is a well‐known feature of plant stress responses and occurs following immune activation and cell death (Atkinson *et al*., 1985). To monitor Rx1‐induced electrolyte leakage over time, agroinfiltrated *Rx1:4xHA *leaf discs were harvested, rinsed in water and placed in water in 96‐well plates and induced with DEX. A hand‐held device (Horiba B‐173 Twin, Kyoto, Kyoto Prefecture, Japan) was used to monitor conductivity over time (0–4 hpda). Conductivity increased rapidly in *CP106 Rx1:4xHA* samples, but not in the *CP105* control. Within 2 hpda, ion leakage was significantly different between CP105 and CP106 samples (Fig. 4A). To test the effect of an altered subcellular localization of Rx1 on this immune output, WT plants were transformed with NLS‐ or NES‐Rx1 variants in combination with DEX‐inducible CP constructs (Fig. 4B). Both Rx1‐NLS and Rx1‐NES specifically induced ion leakage when co‐expressed with CP106, but the rate of electrolyte leakage was significantly slower than that in the CP106‐Rx1‐GFP control (Fig. 4B). Leakage induced by the NES/NLS constructs was less than half that induced by Rx1‐GFP at 1, 2 and 3 hpda. However, whereas at 4 h, Rx1‐NES‐induced leakage was still significantly lower, the conductivity of Rx1‐NLS was indistinguishable from that of Rx1‐GFP (Wilcoxon test, *P* > 0.05) (Fig. 4B). These differences are not caused by variability in Rx1 protein accumulation, as both the NLS and NES fusions accumulate to similar levels to the non‐modified Rx1‐GFP fusion (Fig. S3). Rx1‐GFP activation increased electrolyte leakage at 1 hpda, whereas Rx1‐NES and Rx1‐NLS activations led to a slower increase in leakage, becoming apparent at 2 hpda. These results show that electrolyte leakage following Rx1 activation commences at 1 hpda, and that Rx1‐GFP‐induced leakage relies on Rx1 being able to localize to both the cytosol and the nucleus, as targeted NES and NLS variants induce slower and reduced rates of leakage. An exclusive nuclear localization of Rx1 slows the progression of ion leakage to a lesser extent than nuclear exclusion, which results in electrolyte leakage that trails Rx1‐GFP‐induced leakage at 4 hpda.

### Autofluorescence as a proxy for immune responses and cell integrity

Here, we used a stain‐free method to measure the progression of the immune response in leaf discs based on autofluorescence. A microscopy‐based study by Pietraszewska‐Bogiel *et al*. (2013) showed that, following immune activation, autofluorescence in the far‐red spectrum is reduced, whereas blue light‐excited autofluorescence is increased. We monitored the autofluoresence spectra to study the onset and progression of immune responses using a 96‐well plate format. Autofluorescence measurements were performed by placing leaf discs, sampled from leaves agroinfiltrated with Rx1‐ and CP‐containing constructs, in individual wells of a 96‐well plate containing water and DEX. Hourly measurements were performed using a microplate reader (BioTek) deploying two different excitation and emission spectra, namely 430/530 nm and 480/690 nm (excitation/emission).

**Figure 5:**
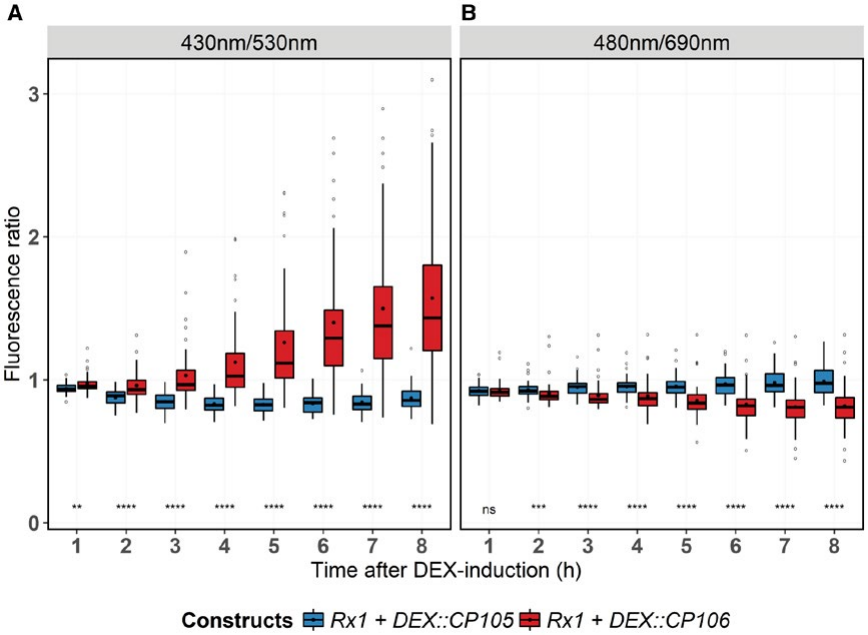
Rx1 activation alters autofluorescence. (A) Changes in autofluorescence at 530 nm in *Rx1::4HA *leaf discs after dexamethasone (DEX) treatment to induce the expression of coat protein (*CP*) constructs. Excitation was at 480 nm; data are depicted in a box‐and‐whisker plot. (B) Changes in autofluorescence at 690 nm after excitation with 480‐nm light. Ratios were obtained by dividing the obtained value for each leaf disc at the different time points by that measured at time point 0. Statistical analysis using Wilcoxon non‐parametric test: ns, no significant difference; **P < 0.01; ***P < 0.001; **** *P* < 0.0001. In both panels, each column consists of values from 47 measurements (15–16 technical replicates of three individual leaves).

After DEX application, autofluorescence at 530 nm increased in Rx1‐CP106‐expressing leaf discs relative to discs producing non‐recognized CP105. A significant difference was apparent at 1 hpda (Fig. 5A). When measuring at 690 nm, significant differences between controls and samples with activated Rx1 were observed after the 2‐h time point (Fig. 5B). Remarkably, an initial general decrease in autofluorescence is found at both wavelengths, as ratios are set to unity at time point 0 (Fig. 5A,B). This decrease disappears gradually over time in CP105‐expressing samples, but not in CP106‐expressing samples.

## Discussion

**Figure 6:**
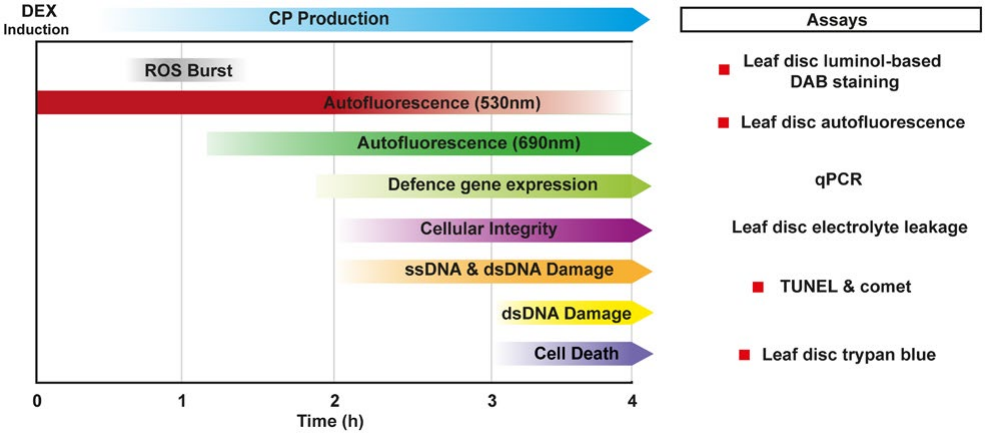
Timing of cellular immune responses following dexamethasone (DEX) induction of *CP106* in *Rx1* *Nicotiana benthamiana. *Graphical summary depicting the chronology of the different immune outputs in a 4‐h timeline following DEX treatment. The developed assays to monitor these outputs are depicted.

We have shown that Rx1 activation induces distinct and phased immune outputs. When taking all data together, a timeline of the various outputs following Rx1 activation can be constructed (Fig. 6). Autofluorescence at 530 nm and ROS production start to increase at <1 hpda, but, unlike ROS, which peaks at 1 hpda, autofluorescence continues to increase (Fig. 5). Ion leakage starts to increase after 1 h, signifying a loss of cellular integrity (Fig. 4). This is in line with the observed reduction in autofluorescence at 690 nm, indicating photosystem collapse. After 2 h, an increase in DNA damage, revealed by both comet and TUNEL assays, becomes apparent and continues over the subsequent hours (Fig. 3). The transcript of *HIN1*, a marker for the onset of cell death, is increased in Rx1‐activated samples at 2 hpda, whereas, after 4 h, transcripts of *PR1‐a*, *LOX*, *ERF1* and *AOX1B* are up‐regulated (Fig. 1A). This finding is relevant as these transcripts mark distinct signalling pathways: *PR1‐a* is a marker for the salicylate‐mediated defence pathway, *AOX1B* can be up‐regulated by salicylate and by endogenous ROS production, *LOX* is a marker for jasmonate‐mediated defence, and *ERF1* is a marker for the ethylene‐mediated defence pathway. It has been reported that, during effector‐triggered immunity (ETI), SA and jasmonate signalling pathways are both activated (van den Berg *et al*., 2018; Liu *et al*., 2016). SA accumulation during ETI promotes the production of jasmonate, allegedly to provide protection against necrotrophic pathogens feeding on dead cells that result from ETI (Liu *et al*., 2016). Our data are in line with this finding, thereby validating our experimental system.

DAB staining and trypan blue staining reveal that both Rx1‐induced ROS production and cell death first appear in single cells and in patchy spots in the infiltrated areas (Figs 1D, 2A and S5, see Supporting Information). These asynchronous effects could be attributed to stochastic variations in the expression levels of the *CP *or *Rx1 *transgene, resulting in differences in sensitivity of the individually transformed cells (Raj and van Oudenaarden, 2008). This heterogeneity in response might also contribute to the relatively wide ROS peak following Rx1 activation (Fig. 2B,C). After DEX application to *Rx1*‐*DEX::CP106* leaf material, ROS production was initiated after about 45 min and lasted for about 1 h, reaching a maximum around 1 hpda (Fig. 2). Rx1‐triggered ROS production is delayed and less confined compared with flg22‐triggered ROS production, which shows a sharp and defined burst peaking around 25 min after treatment (Chinchilla *et al*., 2007; Li *et al*., 2014). Unlike previous reports on NLR activation, Rx1‐induced ROS production was not sustained for several hours (Kadota *et al*., 2015). This difference is probably a result of synchronised Rx1 activation using CESSNA, as opposed to the non‐synchronized activation induced by pathogen‐mediated effector delivery. In fact, our results show that NLR immune activation triggers a defined ROS burst, which is comparable with that induced by flg22. It has been reported that NLR activation gives rise to a biphasic ROS burst, but, given the onset of cell death within a few hours after Rx1 activation, this seems unlikely to happen in our set‐up (Lamb and Dixon, 1997). Future studies using an NLR that triggers ‘weaker’ or ‘slower’ responses could reveal such a biphasic ROS burst. Rx1‐NLS triggers ROS production, although to a lesser degree than non‐modified Rx1 (Figs 2B,C and 4). As Rx1 is activated by CP106 in the cytosol (Slootweg *et al*., 2010), it implies that a small cytosolic Rx1 fraction suffices to activate the ROS‐producing systems. Targeting Rx1 to the cytosol makes Rx1‐NES ‘trigger‐happy’, as elevated ROS levels were measured in the absence of the CP. These elevated ROS levels were further induced in the presence of non‐recognized CP105. These findings propose a localization for the ROS‐producing systems in the cytosol, which is analogous to Arabidopsis RPM1 (also a coiled‐coil NBS‐LRR), where ROS production is mediated by plasma membrane‐localized Respiratory Burst Oxidase Homolog protein D and, to a lesser extent, by Respiratory Burst Oxidase Homolog protein F activity (Torres *et al*., 2002), similar to FLS2 (Chinchilla *et al*., 2007).

Rx1‐NES and Rx1‐NLS variants trigger ROS production, even in combination with CP105 for the Rx1‐NES variant. However, cell death is not activated by CP105‐Rx1‐NES and progresses more slowly for CP106‐Rx1‐NES than with non‐modified Rx1. This suggests that either these responses are uncoupled, implying that cell death is not a consequence of ROS production, or that a ROS threshold must be exceeded for cell death to be executed. Rx1‐NLS triggers the same rate of electrolyte leakage as non‐modified Rx1‐GFP at 4 hpda. When electrolyte leakage is seen as a proxy for the loss of cellular integrity and cell death, these findings support the hypothesis that Rx1‐mediated cell death is initiated in the nucleus, the rationale being that this response is affected to a lesser extent than the ROS response that is triggered by nuclear sequestered Rx1. The finding that CP105 has the potential to weakly activate Rx1 is also reflected in the comet assay, where slightly increased levels of DNA damage are seen in the CP105 samples (Fig. 3B,C). Like the comet assay, the TUNEL assays revealed DNA damage after Rx1 activation (Fig. 3). TUNEL assay data mimic those of the comet assay using AN buffer, as both detect dsDNA and ssDNA damage at 2 hpda (Fig. 3). The fact that DNA damage is detected at an earlier time point for AN buffer (2 hpda) than for NN buffer (3 hpda), which monitors only dsDNA damage, implies that ss breaks precede ds breaks (Fig. 3B,C). If the apparent difference in timing of DNA damage with the TUNEL and comet assays is genuine, it implies that DNAse‐mediated oligonucleosomal DNA fragmentation associated with cell death is preceded by single‐stranded nicks (van Baarlen *et al*., 2004). Rx1, as the tomato NLR I‐2, binds and bends DNA *in vitro* and *in vivo* (Fenyk *et al*., 2015, 2016). Moreover, DNA binding has been reported for several other NLRs (Le Roux *et al*., 2015; Rodriguez *et al*., 2018; Sarris *et al*., 2015). Combining these findings with our data hints at the exciting possibility that DNA binding by Rx1 might induce DNA damage as part of NLR‐triggered immune signalling. To further assess the potential role of the DNA damage machinery in immunity, quantitative RT‐PCR was employed to monitor the expression of DNA damage repair genes following Rx1 activation by CP106. However, within 4 h after DEX treatment, no induction was found of the *PARP1*, *PARP2‐2 *or *Ku70* genes (Fig. S1B). Therefore, although DNA damage occurs rapidly following Rx1 activation, DNA damage repair genes are not transcriptionally regulated in response to this damage. In future studies, histochemical analyses, such as immunostaining of phosphorylated histone‐variant gamma H2Ax, could be used to monitor the location of the DNA damage in the nucleus. The final outcome of Rx1 activation by CP106 is complete collapse of the infiltrated sector after 24 h, as shown by trypan blue staining of whole leaves (Fig. 1D), but, at 4 hpda, there are already significant differences in cell death between CP105 and CP106 samples (Fig. S5).

To conclude, our work has revealed a landscape of Rx1‐triggered responses. In future studies, our approach can be used to compare the ability of various Rx1 variants to activate distinct immune outputs, and possibly decouple responses from one another. The CESSNA toolbox can serve as a generic and universal toolbox for the investigation of other immune receptors and their cognate effectors, as many NLRs, (e.g. I‐2 and Mi1.2 from tomato, MLA10 from barley, to name a few) have been shown to be functional in *N. benthamiana* (Bai *et al*., 2012; Houterman *et al*., 2009; van Ooijen *et al*., 2008). In addition, the system can be readily adjusted to monitor immune outputs of non‐NLR immune receptor–elicitor pairs. It will be interesting to determine the quantitative and qualitative differences between various immune receptors and relate them to their ability to halt pathogen ingress.

## Experimental Procedures

### Plant lines and *Agrobacterium*‐mediated transformation of *N. benthamiana*

WT and transgenic *Rx1:4xHA* *N. benthamiana* were used and grown in long‐day conditions in a climate chamber (22 °C, 70% humidity, 11 h/13 h light/dark).* Agrobacterium*‐mediated transformation was performed on the youngest fully expanded leaves (Ma *et al*., 2012).

### CP and Rx1 constructs

The pTA7002 vector was used to create DEX‐inducible CP constructs (Aoyama and Chua, 1997). CP105 and CP106 were amplified from the pRAP35::CP105 and pRAP35::CP106 vectors and cloned into pTA7002 using the *Xho*I site (Table S2, see Supporting Information) (Slootweg *et al*., 2010). pBIN+ vectors with Rx1 NES and NLS fusions were used as described previously (Slootweg *et al*., 2010).

### Western blotting

For western blot analysis, four leaf discs were sampled from agroinfiltrated areas with a biopsy puncher [Ø = 6 mm; World Precision Instruments (WPI), Sarasota, FL, USA] and placed in 1 mL of 20 μm DEX, 0.01% Silwet R‐77 in Milli‐Q H_2_O. At the desired time points, leaf discs were briefly dried on absorbent paper and ground in liquid nitrogen. Homogenized leaves were incubated in 30 μL of extraction buffer [50 mm Tris‐HCl, pH 6.8, 2% sodium dodecylsulfate (SDS), 2 mm dithiothreitol (DTT) and 1 × protease inhibitors (Roche, Basel, Switzerland)] for 30 min at room temperature and centrifuged for 5 min at 13 000 ***g***. The supernatant was mixed 5 : 1 with 6 × Laemmli buffer (375 mm Tris‐HCl, pH 6.8, 6% SDS, 20% v/v glycerol, 0.03% bromophenol blue and 100 mm DTT) and boiled for 5 min. Total proteins were separated by 15% sodium dodecylsulfate‐polyacrylamide gel electrophoresis (SDS‐PAGE) and (semi‐dry) blotted onto polyvinylidene fluoride (Immobilon‐P, MilliporeSigma, Burlington, MA, USA) membranes. PVX‐CP was detected using a PVX‐specific polyclonal antibody (diluted 1 : 3000) (ref. 110411, Bioreba, Reinach, Switzerland), followed by incubation with horseradish peroxidase (HRP)‐conjugated goat anti‐rabbit immunoglobulin G (IgG) secondary antibody (diluted 1 : 10 000) (ref. 31460, ThermoFischer Scientific, Waltham, MA, USA). Secondary immunoglobulins were visualized using home‐made ECL solution containing 2.5 mm luminol, 0.4 mm *p*‐coumaric acid, 100 mm Tris‐HCl, pH 8.5, and 0.018% H_2_O_2_. Incubation of both primary and secondary antibodies was performed in Tris‐buffered saline with 0.05% Tween‐20 (TBST), followed by three rinses of 10 min in TBST. Equal protein loading was confirmed for the samples by Ponceau S staining of the membranes.

### Gene expression analysis

For gene expression analysis, 15 leaf discs were sampled from agroinfiltrated areas with a biopsy puncher (Ø = 6 mm, WPI) and placed in 1 mL of 20 μm DEX, 0.01% Silwet R‐77 in Milli‐Q H_2_O. At the desired time points, leaf discs were dried on absorbent paper and ground in liquid nitrogen. Total RNA was extracted using TRIzol LS reagent (ThermoFischer Scientific, Waltham, MA, USA). The RNA was treated with DNase (ThermoFischer Scientific) according to the supplier’s protocol, and RNA concentrations were determined by measuring the absorbance at 260 nm [Abs(260)] on a Nanodrop (ThermoFischer Scientific). cDNA was synthesized from 1 μg of total RNA using RevertAid H reverse transcriptase and Oligo‐dT (Eurofins) in the presence of the RNAse inhibitor Ribolock (ThermoFischer Scientific), following the supplier’s protocol, and diluted 10 times in Milli‐Q H_2_O.

Quantitative RT‐PCR was performed with a model 7500 Real Time PCR system (ThermoFischer Scientific) and Solis Biodyne (Tartu, Estonia) BioDyne 5× HOT FIREPol Eva Green qPCR Mix Plus (ROX) on 1 μL of diluted cDNA. The Ct values were corrected for primer efficiencies; the gene expression level was normalized using the reference genes EF1α and PP2A, and was calculated using qBASE+ (Biogazelle, Zwijnaarde, Belgium).

Semi‐quantitative RT‐PCR (25 cycles, annealing temperature of 60 ºC) was performed on 1 μL of diluted cDNA using DreamTaq DNA Polymerase (ThermoFischer Scientific) following the supplier’s protocol. Primers adapted from other studies (Liu *et al*., 2012; Peiman and Xie, 2006; del Pozo *et al*., 2004; Zhang *et al*., 2015; Zhu *et al*., 2015) and newly designed for the expression analysis are listed in Table S2.

### Ion leakage assay

Leaf discs were taken from agroinfiltrated leaves with a biopsy puncher (Ø = 5 mm, WPI). Leaf discs from one infiltration zone were collected and placed in 1 mL of Milli‐Q H_2_O (e.g. in a Greiner Bio‐one Cellstar six‐well plate, Kremsmuenster, Austria). After harvesting, the leaf discs were transferred to a 48‐well plate (Greiner Bio‐one Cellstar 48‐well plate). Two leaf discs were placed in a well, which contained 100 µL of Milli‐Q H_2_O. At least three technical replicates were present per infiltration zone. Afterwards, 100 µL of DEX solution was added [DEX solution; 40 µm DEX (VWR International), 0.01% Silwet R‐77 in Milli‐Q H_2_O]. The plate was carefully and briefly horizontally shaken. To measure the conductivity, 50 µL of the leaf disc’s bathing solution was pipetted onto the sensor of a Horiba B‐173 Twin Conductivity Meter (Horiba Scientific) and pipetted back into the designated well after measurement. This was repeated for all wells and time points.

### Plate reader ROS burst assay

Leaf discs were taken from agroinfiltrated leaves with a biopsy puncher (Ø = 5 mm, WPI). Leaf discs from one infiltration zone were placed in 1 mL of Milli‐Q H_2_O and incubated overnight at room temperature (in a six‐well plate). Leaf discs were then transferred to a white 96‐well plate (Perkin‐Elmer Optiplate 96, Waltham, MA, USA), one disc per well, containing 100 µL of Milli‐Q H_2_O. Subsequently, 100 µL of DEX/luminol solution was added [40 µm DEX, 500× diluted peroxidase (Sigma‐Aldrich, P6782, St. Louis, MO, USA), 500× diluted luminol (Sigma‐Aldrich, 09253) and 0.01% Silwet R‐77 in Milli‐Q H_2_O] to each well. The plate was placed in a BioTek Synergy H1 Hybrid multi‐mode microplate reader (BioTek) and luminescence was measured per well for 105 min at 3–4‐min intervals.

### DAB staining

Four‐week‐old *Rx1:4xHA* plants were agroinfiltrated with *DEX::CP105* and *DEX::CP106. *Two days after transformation, sectors were infiltrated with DAB staining solution (1 mg/mL 3,3′‐diaminobenzidine‐4HCl in 20 mm Na_2_HPO_4_). After infiltration, leaves were induced by brushing 20 µm DEX in 0.01% Silwet and sampled at different time points. Leaves were de‐stained by boiling for 15 min in ethanol–glycerol–acetic acid (3 : 1 : 1). Leaves were mounted in ethanol–glycerol–water (1 : 1 : 2) in Petri dishes and photographed using an Epson Perfection V750 flatbed scanner (Epson, Suwa, Nagano Prefecture, Japan).

### Comet assay

The comet assay was performed according to Menke *et al*. (2001) with modifications. Infiltrated leaf sections (about one‐quarter of the total surface per leaf) were cut and flash frozen in liquid N_2_. Electrophoresis was performed in a standard electrophoresis tank (Bio‐rad, Hercules, CA, USA) at 31 V as described, but the duration was extended to 7 min (AN buffer) and 10 min (NN buffer). Slides were stained with DAPI (1 µg/mL) solution and scored using an EVOS FL Cell Imaging System (Thermo Fisher Scientific). Photographs were analysed using CASPlab software (version 1.2.2) from Casplab.org (Konca *et al*., 2003).

### TUNEL assay

Half of an infiltrated *N. benthamiana* leaf was finely chopped in a Petri dish in 500 µL of ice‐cold Nuclear Isolation Buffer (120 mm KCl, 30 mm NaCl, 1 mm spermidine, 0.3 mm spermine, 680 mm sucrose and 30 mm Tris‐HCl, pH 7.5). Approximately 250 µL of nuclei‐containing liquid was transferred to a 1.5‐ml Eppendorf (Hamburg, Germany) tube using a cut 1000‐µL pipette tip which was placed on the surface of the suspension to preferentially harvest nuclei and not cell debris. The nuclei were harvested following a 1‐min centrifugation step at 2600 rpm (582g) and resuspended in 250 µL of cold PBS buffer (137 mm NaCl, 2.7 mm KCl, 10 mm Na_2_HPO_4_ and 1.8 mm K_2_HPO_4_, pH 7.5). After spinning, the supernatant was removed and nuclei were fixed by adding 400 µL of ice‐cold methanol and glacial acetic acid (ratio 3 : 1) at 4 °C for 15 min. The nuclei were pelleted and washed twice in 300 µL of cold PBS buffer. After removing the supernatant, 25 µL of TUNEL reaction mix was added (2.5 µL enzyme solution in 22.5 µL label solution, Roche *In Situ* Cell Death Detection Kit, fluorescein). The sample was subsequently incubated for 2 h in the dark at 37 °C, with occasional agitation. Next, nuclei were pelleted, washed twice in 300 µL of ice‐cold PBS buffer and resuspended in 80 µL of PBS and 10 µL of DAPI (1 µg/mL). Per sample, 25 µL was placed in a single well of a black 96‐well plate (Greiner Bio‐one Microplate‐96 µClear), and 25 µL of PBS was added. The integrity of the nuclei was checked by mounting 5 µL of the remaining sample on a microscopy slide with visual observation using an EVOS FL Cell Imaging System. A BioTek Synergy H1 Hybrid multi‐mode microplate reader (BioTek) was used to measure the fluorescence of both the DAPI and fluorescein signals. For DAPI, 370‐nm excitation and 455‐nm emission were used; for fluorescein, 480‐nm excitation and 525‐nm emission were used. Ratios of fluorescein : DAPI were calculated to normalize the variation in the amount of DNA.

### Trypan blue staining

Trypan blue staining was applied on whole leaves (Ma *et al*., 2012). Leaves were boiled for 5 min in a 1 : 1 mixture of 96% ethanol and staining solution [100 mL lactic acid, 100 mL phenol, 100 mL glycerol, 100 mL H_2_O and 100 mg trypan blue (Sigma‐Aldrich, 93590)]. Leaves were de‐stained overnight in 2.5 g/mL chloral hydrate in water (Ma *et al*., 2012). Leaves were spread out in a Petri dish and photographed using an Epson Perfection V750 flatbed scanner (Epson, Suwa, Nagano Prefecture, Japan).

### Autofluorescence assay

Leaf discs were sampled from infiltrated leaves with a biopsy puncher (Ø = 6 mm, WPI). Leaf discs from one infiltration zone were placed in 1 mL of Milli‐Q H_2_O (e.g. in a six‐well plate). The leaf discs were transferred to a black 96‐well plate (PerkinElmer Optiplate‐96), one disc per well, with the abaxial side of the leaf upwards, containing 100 µL of Milli‐Q H_2_O. CP expression was induced by the addition of 100 µL of DEX solution [DEX solution: 40 µm DEX (VWR International), 0.01% Silwet R‐77 in Milli‐Q H_2_O]. Plates were placed under fluorescent lighting and measured at set time points using a BioTek Synergy H1 Hybrid multi‐mode microplate reader (BioTek).

To detect autofluorescence associated with phenolic compounds, excitation was at 400 nm and emitted light was collected at 530 nm; loss of autofluorescence associated with chlorophyll was measured using excitation at 480 nm and emission at 690 nm. Both spectral combinations were set using the monochromator of the BioTek Synergy microplate reader.

### Plots and figures

Graphs and plots were generated using the ‘R’ language for statistical computing and graphics (www.r-project.org) and RStudio software (RStudio, 2015). Scripts for the generation of the figures, including the statistical analyses, are available at https://github.com/MolPlantPathology/. Adobe Illustrator 2017 CC was used for image creation and editing (Adobe Inc., San Jose, CA, USA).

## Conflicts of Interest

The authors have no conflicting interests to declare.

## Supporting information


**Fig. S1  **Expression levels of defence and DNA damage‐associated genes after Rx1 activation. (A) Levels of *PR‐1b*, *PR‐2b* and *MAP3ka* transcripts at 0, 2 and 4 h after dexamethasone (DEX) application (hpda) measured using quantitative polymerase chain reaction (PCR). (B) Levels of *PARP1*, *PARP2‐2* and *Ku70* transcripts at 0, 2 and 4 hpda measured using quantitative PCR. Data are the means ± standard error (SE), normalized by *EF1α* and *PP2A* expression. Asterisks indicate significant differences by one‐way analysis of variance (ANOVA) (*P* < 0.0001).

**Fig. S2  **flg22‐induced reactive oxygen species (ROS) production in leaf discs. The production of ROS after application with and without (mock treatment) application of 1 µm of flg22. Leaf discs taken from 4‐week‐old *Nicotiana benthamiana* plants were measured using a luminol‐based assay in an optical plate reader. Signal intensity is represented in arbitrary units (AU). Each value represents 10 technical replicates. The experiment was repeated at least three times with similar results.

**Fig. S3  **Western blot analysis of Rx1 constructs after *Agrobacterium*‐mediated transformation of *Nicotiana benthamiana* leaves. Western blot detecting levels of Rx1 fusion proteins in mock, Rx1‐GFP, Rx1‐NES‐GFP and Rx1‐NLS‐GFP transformed leaves using anti‐green fluorescent protein (anti‐GFP) antibody (top panel). Total protein loading was visualized using Ponceau staining (bottom panel). Blots were probed with horseradish peroxidase (HRP)‐conjugated goat anti‐rabbit immunoglobulin G (IgG) secondary antibody. NES, nuclear export signal; NLS, nuclear localization signal.

**Fig. S4  **Comet assay of dexamethasone (DEX)‐treated samples expressing *DEX::GFP*. Comet assay showing DNA damage in plant nuclei with induced *DEX::GFP* at 2, 4 and 8 h after induction using high‐alkaline (AN) buffer, showing single‐stranded DNA (ssDNA) and double‐stranded DNA (dsDNA) breaks. Nuclei (comets) were counted and visualized using a box‐and‐whisker plot. The number of nuclei per sample is depicted in the box for each sample. Statistical analysis using Wilcoxon non‐parametric test: ns, no significant difference; *P < 0.01.

**Fig. S5  **Rx1‐induced cell death at 4 h after dexamethasone (DEX) induction visualized using trypan blue. Trypan blue visualizes cell death. *Rx1:4xHA* leaves infiltrated with *Agrobacterium tumefaciens *carrying *DEX::CP105* and *DEX::CP106* constructs. Two days after infiltration, the leaves were brushed with 20 µm DEX and stained 4 h later. One leaf was scanned with a white (left) and black (right) background to visualize trypan blue staining of dead cells.

**Table S1  **Overview of comet assay samples. This table shows an overview of the samples and the number of comets (nuclei) that were counted and depicted in Fig. 4.

**Table S2  **Primer list. Name, sequence and description of all primers used in the quantitative polymerase chain reaction (PCR) experiment shown in Fig. 1A.

**Video S1  **Time lapse of Rx1‐triggered cell death. Time lapse movie showing Rx1‐triggered tissue collapse after dexamethasone (DEX)‐induced CP106 expression (right bottom sector, marked with ‘6’ on the leaf) and control (top left sector) with CP105 expression. This time lapse was created by photographing the leaf of a 4‐week‐old *Nicotiana benthamiana* Rx1:4xHA plant, transformed with *DEX::CP105* and *DEX::CP106*, every 10 min for 8 h after DEX was brushed onto the leaf surface.
