# Supplementary figures and images for: Activation of immune receptor Rx1 triggers distinct immune responses culminating in cell death after 4 hours
Source: Mol Plant Pathol. 2019 Jan 30;20(4):575–88. doi: 10.1111/mpp.12776 (PMC6637897; doi:10.1111/mpp.12776)

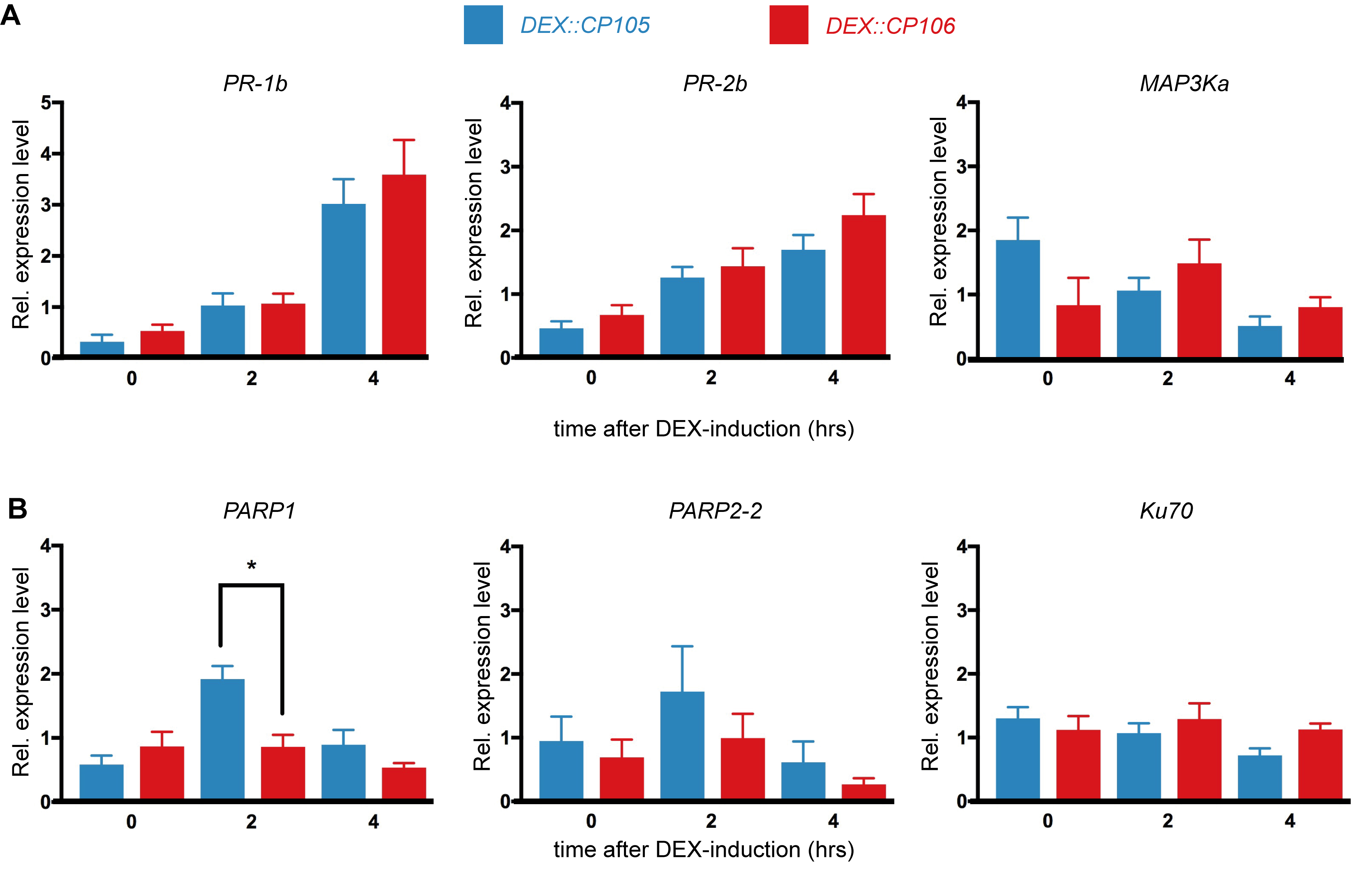

Supplement: Supplementary file 1 — Fig. S1 Expression levels of defence and DNA damage‐associated genes after Rx1 activation. (A) Levels of PR‐1b, PR‐2b and MAP3ka transcripts at 0, 2 and 4 h after dexamethasone (DEX) application (hpda) measured using quantitative polymerase chain reaction (PCR). (B) Levels of PARP1, PARP2‐2 and Ku70 transcripts at 0, 2 and 4 hpda measured using quantitative PCR. Data are the means ± standard error (SE), normalized by EF1α and PP2A expression. Asterisks indicate significant differences by one‐way analysis of variance (ANOVA) (P < 0.0001). [file MPP-20-575-s001.tif]

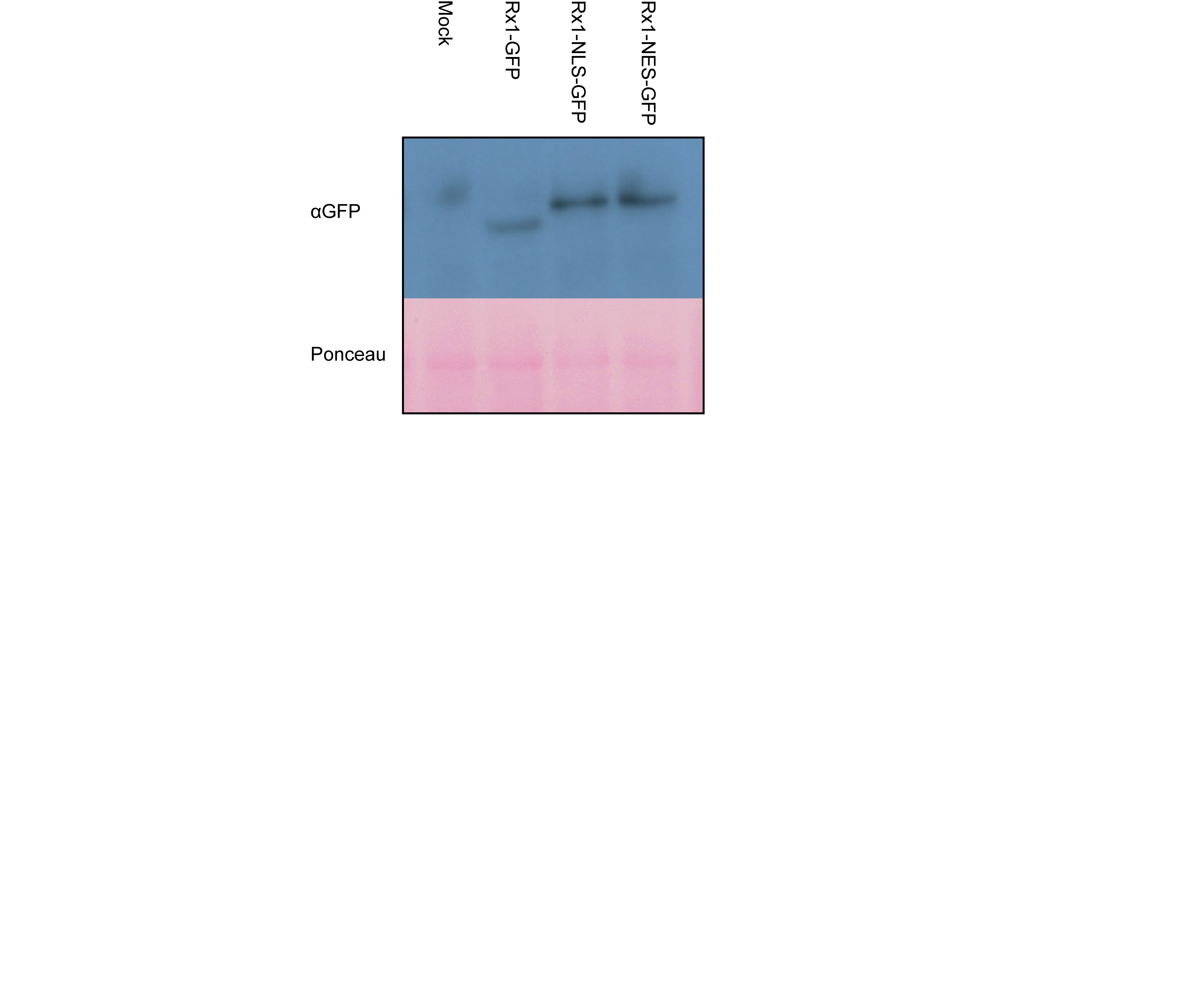

Supplement: Supplementary file 3 — Fig. S3 Western blot analysis of Rx1 constructs after Agrobacterium‐mediated transformation of Nicotiana benthamiana leaves. Western blot detecting levels of Rx1 fusion proteins in mock, Rx1‐GFP, Rx1‐NES‐GFP and Rx1‐NLS‐GFP transformed leaves using anti‐green fluorescent protein (anti‐GFP) antibody (top panel). Total protein loading was visualized using Ponceau staining (bottom panel). Blots were probed with horseradish peroxidase (HRP)‐conjugated goat anti‐rabbit immunoglobulin G (IgG) secondary antibody. NES, nuclear export signal; NLS, nuclear localization signal. [file MPP-20-575-s003.tif]

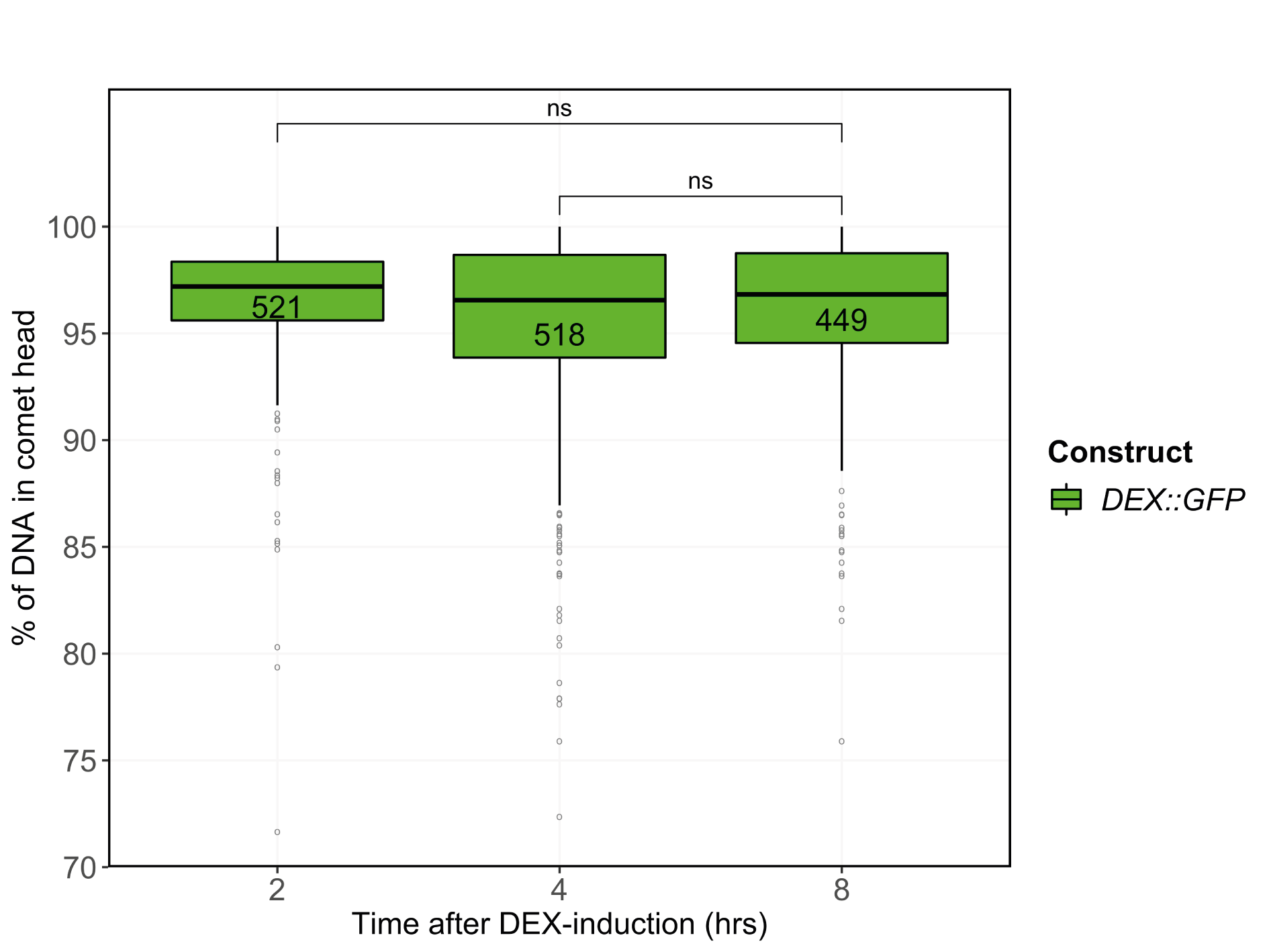

Supplement: Supplementary file 4 — Fig. S4 Comet assay of dexamethasone (DEX)‐treated samples expressing DEX::GFP. Comet assay showing DNA damage in plant nuclei with induced DEX::GFP at 2, 4 and 8 h after induction using high‐alkaline (AN) buffer, showing single‐stranded DNA (ssDNA) and double‐stranded DNA (dsDNA) breaks. Nuclei (comets) were counted and visualized using a box‐and‐whisker plot. The number of nuclei per sample is depicted in the box for each sample. Statistical analysis using Wilcoxon non‐parametric test: ns, no significant difference; *P < 0.01. [file MPP-20-575-s004.tif]

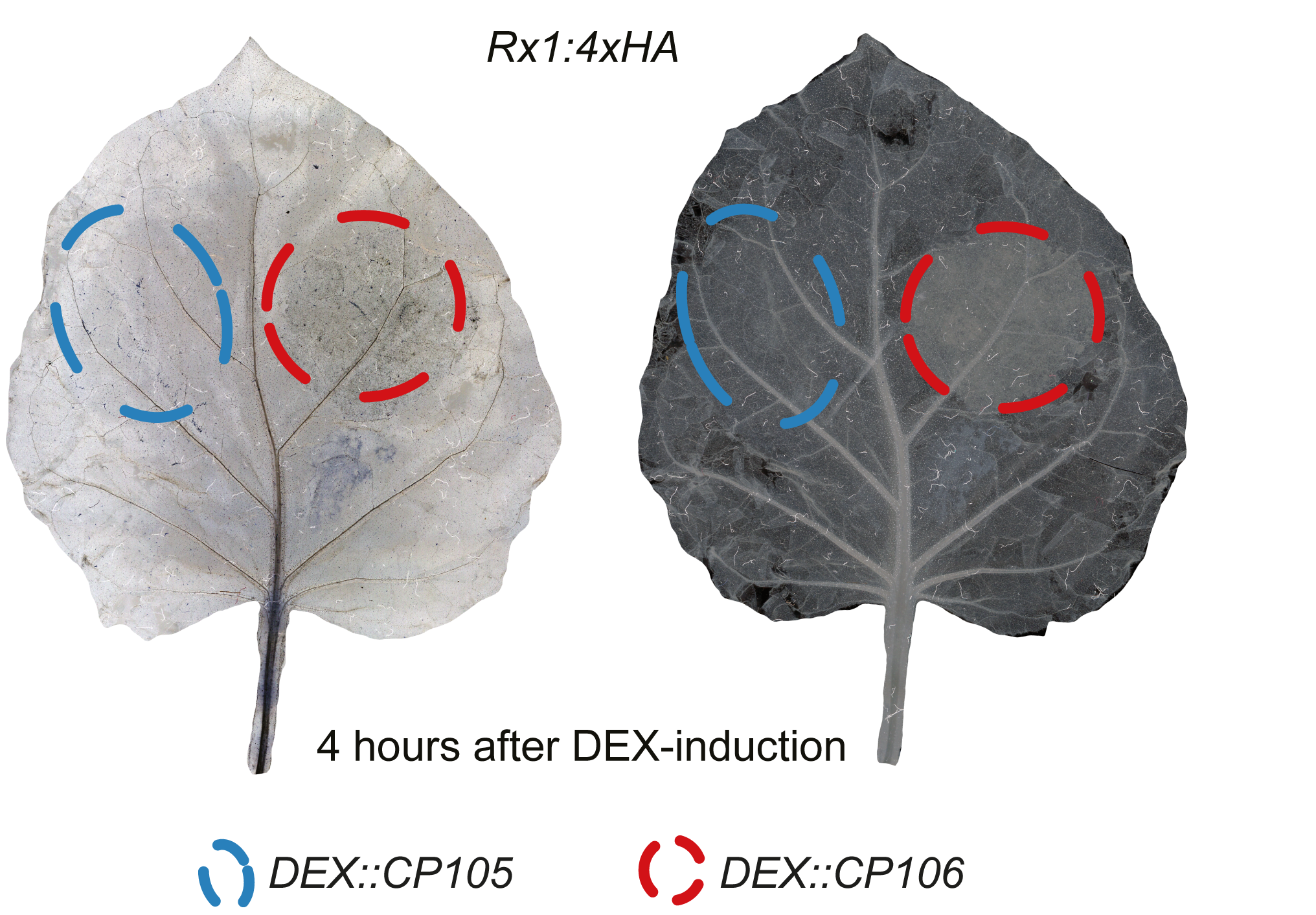

Supplement: Supplementary file 5 — Fig. S5 Rx1‐induced cell death at 4 h after dexamethasone (DEX) induction visualized using trypan blue. Trypan blue visualizes cell death. Rx1:4xHA leaves infiltrated with Agrobacterium tumefaciens carrying DEX::CP105 and DEX::CP106 constructs. Two days after infiltration, the leaves were brushed with 20 µm DEX and stained 4 h later. One leaf was scanned with a white (left) and black (right) background to visualize trypan blue staining of dead cells. [file MPP-20-575-s005.tif]
