# Supplementary material for: Activation of immune receptor Rx1 triggers distinct immune responses culminating in cell death after 4 hours
Source: Mol Plant Pathol. 2019 Jan 30;20(4):575–88. doi: 10.1111/mpp.12776 (PMC6637897; doi:10.1111/mpp.12776)
Supplement: Supplementary file 6 — Table S1 Overview of comet assay samples. This table shows an overview of the samples and the number of comets (nuclei) that were counted and depicted in Fig. 4. [file MPP-20-575-s006.docx]

| **Time (hours)** | **Construct** | **Experiment** | **# of comets** |
| --- | --- | --- | --- |
| 0 | CP105 | AN | 284 |
| 0 | CP105 | NN | 177 |
| 0 | CP106 | AN | 289 |
| 0 | CP106 | NN | 185 |
| 1 | CP105 | AN | 562 |
| 1 | CP105 | NN | 236 |
| 1 | CP106 | AN | 676 |
| 1 | CP106 | NN | 248 |
| 2 | CP105 | AN | 401 |
| 2 | CP105 | NN | 260 |
| 2 | CP106 | AN | 975 |
| 2 | CP106 | NN | 280 |
| 3 | CP105 | AN | 358 |
| 3 | CP105 | NN | 322 |
| 3 | CP106 | AN | 377 |
| 3 | CP106 | NN | 347 |
| 4 | CP105 | AN | 699 |
| 4 | CP105 | NN | 311 |
| 4 | CP106 | AN | 631 |
| 4 | CP106 | NN | 404 |
| 4 | GFP | AN | 345 |

**Table S1. Overview of comet assay samples.** This table shows an overview of the samples and the number of comets (nuclei) that were counted and depicted in Figure 4.
