# Supplementary material for: Activation of immune receptor Rx1 triggers distinct immune responses culminating in cell death after 4 hours
Source: Mol Plant Pathol. 2019 Jan 30;20(4):575–88. doi: 10.1111/mpp.12776 (PMC6637897; doi:10.1111/mpp.12776)
Supplement: Supplementary file 7 — Table S2 Primer list. Name, sequence and description of all primers used in the quantitative polymerase chain reaction (PCR) experiment shown in Fig. 1A. [file MPP-20-575-s007.pdf]

Primer Name	Primer Sequence (5' - 3')	Description
**FP6743-NbPR-1a-F**	TGCCTTCATTTCTTCTTGTC	qRT-PCR
**FP6744-NbPR-1a-R**	GTTATGGGCATCCAAATAGT	A. Goverse WUR, pers. com.
**FP6946-NbLOX-F**	AAAACCTATGCCTCAAGAAC	qRT-PCR
**FP6947-NbLOX-R**	ACTGCTGCATAGGCTTTGG	Zhang et al.,2012
**FP6970-NbHIN1-F**	CATTAGTTCTATGGCTCGTT	qRT-PCR
**FP6971-NbHIN1-R**	ATGGCATCTGGTTTCCT	Zhu et al., 2015
**FP8369-NbPP2A-F**	GACCCTGATGTTGATGTTCGCT	qRT-PCR and RT-PCR
**FP8370-NbPP2A-R**	GAGGGATTTGAAGAGAGATTTC	Liu et al., 2015
**FP8371-PVX-CP-F**	CACTGCAGGCGCAACTCC	RT-PCR
**FP8372-PVX-CP-R**	GTCGTTGGATTGYGCCCT	Peiman & Xie 2006
**FP8391-NbEF1α-F**	AGCTTTACCTCCCAAGTCATC	qRT-PCR and RT-PCR
**FP8392-NbEF1α-R**	AGAACGCCTGTCAATCTTGG	Liu et al., 2015
**FP6745-NbMAP3Ka-F**	AGGAAATGGCGATAATAGGT	qRT-PCR
**FP6746-NbMAP3Ka-R**	AAAGGTGATGATGTGGTAGT	del Pozo et al., 2004
**FP6972-NbAOX1b-F**	CTTCTTCAACGCCTATT	qRT-PCR
**FP6973-NbAOX1b-R**	CAGCCCTAACAACCAA	Zhu et al., 2015
**FP6944-NbERF1-F**	GCTCTTAACGTCGGATGGTC	qRT-PCR
**FP6945-NbERF1-R**	AGCCAAACCCTAGCTCCATT	Zhang et al.,2012
**FP6976-NbKu70-F**	AATGCCTCTGAGATGCCGTC	qRT-PCR
**FP6977-NbKu70-R**	GGTCCCAACAGATACCCAGC	This study
**FP6653-NbPR-1b-F**	GTTGGTGTTGGCCCCATGAC	qRT-PCR
**FP6654-NbPR-1b-R**	CACATTTTTACGGCACCAGCG	Zhang et al.,2012
**FP8594-NbPR-2b-F**	AGGTGTTTGCTATGGAATGC	qRT-PCR
**FP8595-NbPR-2b-R**	TCTGTACCCACCATCTTGC	Zhang et al.,2012
**FP8596-NbPARP1-F**	CAGGATATATGTTTGGAAAGGGAC	qRT-PCR
**FP8597-NbPARP1-R**	AAGAATTGCAACTTTACTTGGGC	This study
**FP6659-PARP2-2-F**	TACATAGTTTACAACGTGGAG	qRT-PCR
**FP6660-PARP2-2-R**	ATACCAAAATATCGGCAACG	This study

Table S2. Primer list. This table provides the name, sequence and description of all primers used in the qPCR experiment shown in Figure 1a.
